# The relationship between job demands, rumination, and burnout among healthcare professionals in Türkiye: a mediation model

**DOI:** 10.1186/s12913-026-15345-w

**Published:** 2026-08-10

**Authors:** Burak Özdemir, Hamit Murat Özcan, Umut Çil, Gökhan Kerse

**Affiliations:** 1 Department of International Trade and Logistics, Kastamonu University, Kastamonu, Türkiye; 2 Department of Business Administration, Eskişehir Osmangazi University, Eskişehir, Türkiye; 3 Department of Business Administration, Karamanoğlu Mehmetbey University, Karaman, Türkiye; 4 Department of Management Information Systems, Kafkas University, Kars, Türkiye

**Keywords:** Workload, Emotional demands, Emotional dissonance, Burnout, Rumination, Conservation of Resources Theory, Public health, PLS-SEM

## Abstract

**Background:**

While burnout has reached alarming levels globally, the psychological processes linking different job demands to burnout remain insufficiently understood. Drawing on Conservation of Resources (COR) theory, this study examines whether rumination is associated with the relationship between job demands (workload, emotional demands, and emotional dissonance) and burnout.

**Methods:**

A cross-sectional design was employed using convenience sampling. Data were collected from 408 healthcare professionals (physicians, nurses, and other staff) in Türkiye using validated self-report measures. The proposed relationships were analysed using partial least squares structural equation modelling (PLS-SEM).

**Results:**

Among the job demands, workload was positively associated with burnout, with no significant indirect association through rumination. In contrast, emotional demands and emotional dissonance did not show significant direct associations with burnout; however, both variables showed significant indirect associations with burnout through rumination.

**Conclusions:**

The findings suggest that rumination-related cognitive processes play a role in the associations of emotional demands and emotional dissonance with burnout, whereas the association between workload and burnout appears to be more direct. These findings highlight the potential relevance of emotional experiences and rumination in understanding burnout among healthcare professionals.

## Introduction

The mental health of healthcare professionals is of critical importance for the resilience of health systems and the quality of care delivered. Reports by the World Health Organization [1] and recent public health evidence emphasize that burnout, which has reached epidemic proportions among healthcare workers, should not be regarded merely as an individual-level problem; rather, it constitutes a profound public health crisis linked to increased medical errors, reduced care quality, and workforce attrition [2, 3]. In this regard, understanding the mechanisms that deplete the psychological resources of healthcare professionals has become a strategic priority for ensuring the sustainability of healthcare provision and safeguarding overall public health [4, 5].

The healthcare sector constitutes one of the most demanding work environments, characterized by high patient loads, time pressure, and the necessity of sustained emotional labor. Such chronic working conditions represent fundamental systemic factors that increase the risk of burnout among employees [6]. Recent research indicates that burnout levels among healthcare professionals have reached between 40% and 60% in many countries, compromising patient safety and the capacity of health systems to respond effectively to crises [1, 7, 8]. Particularly in the post-pandemic period, the intensification of both workload and emotional burden has significantly elevated levels of depression, anxiety, and burnout, highlighting it as a direct public health concern [7, 9].

One of the most robust theoretical frameworks explaining the relationship between job demands and burnout is the Conservation of Resources (COR) theory. According to this perspective, individuals strive to preserve valuable resources such as time, energy, and psychological resilience; however, high job demands rapidly deplete these resources, leading to stress and ultimately burnout [10, 11]. Contemporary findings further reinforce the relevance of COR theory within a public health context by demonstrating that chronic job demands in healthcare not only result in individual resource depletion but also cause to institutional-level consequences such as absenteeism and disruptions in access to healthcare services [12]. Nevertheless, the “cognitive bridges” through which job demands translate into burnout remain insufficiently clarified in the existing literature. In this study, rumination is examined as a key mechanism in this process.

Sonnentag and Meier [13] argue that studies grounded in COR theory should clearly distinguish static between-person differences and dynamic within-person processes, with particular emphasis on the temporal production of resource loss. Such an approach would enable a more in-depth examination of interactions among resources within the framework of gain and loss cycles [13]. Indeed, this observation aligns with a structural limitation of COR theory that has been noted since its early formulations: the theory provides only a limited account of which resources function as antecedents or consequences of others, and through which micro-psychological mechanisms such transformations occur [14].

Recent research highlights that examining the relationship between job demands and burnout solely as a direct influence is insufficient, and that the cognitive and emotional processes underlying this relationship must also be considered [15–17]. Accordingly, there is a need for a process-oriented and mechanism-focused approach that explicates how job demands lead to burnout. In this context, work-related rumination emerges as a critical cognitive mechanism within between-person processes, facilitating the spillover of work stress into employees’ non-work lives [18, 19]. This study conceptualizes rumination not merely as an outcome variable, but as a “cognitive engine” that sustains the continuity of job demands within the individual’s cognitive system, thereby accelerating the cycle of resource loss. From this perspective, the research aims to uncover the fundamental mechanism that explains under which conditions emotional demands remain a “challenge,” and under which conditions they transform into a resource-depleting “hindrance.”

Previous research has examined rumination as a mediator; however, these studies often relied on other theoretical frameworks [20–22] Consequently, the theoretical mechanism through which different dimensions of job demands contribute to burnout via rumination remains insufficiently explained from the resource loss perspective of COR theory. Similarly, the majority of existing studies fail to adequately account for the differential associations of distinct types of job demands with rumination [23, 24], leaving empirical evidence fragmented regarding the relationships between these dimensions and burnout. This limitation highlights the need for integrative models that simultaneously examine the distinct dimensions of job demands, the mediating role of rumination, and their associations with burnout within a unified theoretical framework. Drawing on COR theory, rumination is argued to deplete executive function resources, generate threat-perception biases, and thereby confine individuals within a self-reinforcing cycle of cognitive and emotional resource depletion [25]. By specifically focusing on workload, emotional demands and emotional dissonance as distinct dimensions of job demands, and by integrating these dimensions with rumination and burnout within a unified COR-based framework, this study aims to advance understanding of the cognitive dynamics underlying resource-loss spirals. In this context, the study examines the associations of workload, emotional demands and emotional dissonance with burnout among healthcare professionals, and tests the potential mediating role of rumination within the COR framework. While a substantial body of research on rumination has focused on general employee samples, the present study provides empirical evidence specific to healthcare professionals, who constitute a high-risk group due to elevated job demands and intense emotional burden. Accordingly, this study addresses both a theoretical and an empirical gap by integrating workload, emotional demands and emotional dissonance with rumination and burnout within the COR framework and by empirically testing the proposed relationships among healthcare professionals.

## Theoretical background and hypotheses

### Conservation of resources (COR) Theory and the selection of job demands

This study is grounded in the COR Theory [10], which emphasizes individuals’ motivation to protect and develop valued resources such as energy, time, social support, and self-efficacy. According to COR theory, stress emerges when these resources are threatened, actually lost, or when the expected returns on invested resources fail to materialize [11]. For healthcare professionals, occupational demands constitute a continuous process of resource expenditure. In this regard [8], conceptualize burnout not merely as a state of fatigue, but as a process of “resilience erosion” resulting from individuals’ inability to replenish their resources under conditions of chronically high job demands.

Although healthcare delivery involves a wide range of job demands, the present study specifically focuses on “proximal stressors” that directly target healthcare professionals’ cognitive and emotional capacities [26]. This focus is consistent with the COR theory’s principle of the “primacy of resource loss,” as the resources most rapidly depleted in everyday work—namely energy, attention, and emotional self-regulation capacity—are directly implicated in such demands. More macro-level demands reflecting organizational change processes (e.g., workplace transformations) are excluded from the scope of this study, as the research question is constructed around between-person mechanisms in COR theory, emphasizing the daily, interaction-based demands that healthcare professionals encounter in patient and team contexts and that intensify immediate resource depletion.

Within this framework, the selection of workload, emotional demands, and emotional dissonance as focal dimensions is grounded in the fact that these three variables represent the primary “energy drainers” that trigger resource loss spirals in healthcare settings [27, 28]. While workload depletes physical and cognitive energy, emotional demands and emotional dissonance directly target their limited emotional resources. Emotional dissonance, in particular, generates a substantial “cognitive cost” arising from the discrepancy between genuinely felt emotions and organizational requirements, severely exhausting employees’ constrained emotional self-regulation resources [29, 30]. Taken together, these three jobs demands function as core stressors that activate the health impairment process proposed by COR theory [31].

### Jobdemands and burnout

Job demands refer to the physical, cognitive, and emotional requirements that employees encounter while performing their work [32]. From COR perspective, although individuals attempt to meet these demands by expending their existing resources, the persistence and intensity of such demands may exceed the capacity for resource recovery, ultimately leading to burnout. Factors such as high workload, time pressure, and elevated responsibility can deplete limited resources such as energy and attention. According to COR theory, individuals experience stress when they lose resources, and when this loss persists, it may evolve into burnout [10]. The literature widely acknowledges job demands as one of the most significant predictors of burnout [26, 33–36]. Recent meta-analytic evidence further confirms that the prevalence of burnout among healthcare professionals has reached 48%, with excessive job demands identified as the most powerful underlying predictor [8].

The healthcare sector represents one of the most demanding work environments due to high patient loads, intense work pace, and emotionally challenging situations [37]. Such conditions can deplete employees’ physical and psychological resources, thereby increasing the risk of burnout. Workload refers to the volume of tasks that healthcare professionals must complete under time pressure and directly consumes energy resources [38]. Emotional demands encompass processes such as witnessing patients’ suffering and the necessity of empathizing with them, whereas emotional dissonance refers to the resource loss arising from the discrepancy between employees’ genuine feelings and the emotions they are expected to display by the organization [28].

Within the COR framework, existing research demonstrates that the high workload, time pressure, and emotional demands faced by healthcare professionals lead to resource depletion, which in turn increases levels of burnout—another form of resource loss [34, 38–41]. Accordingly, all three types of job demands are expected to erode healthcare professionals’ mental well-being and trigger burnout [7]. Based on these considerations, the following hypotheses are proposed:

#### H_1_:

Workload has a direct and positive effect on healthcare professionals’ levels of burnout.

#### H_2_:

Emotional demands have a direct and positive effect on healthcare professionals’ levels of burnout.

#### H_3_:

Emotional dissonance has a direct and positive effect on healthcare professionals’ levels of burnout.

### Job demands and rumination

Within the framework of COR theory, the relationship between job demands and rumination can be explained through the depletion of resources (e.g., energy, time, cognitive capacity), individuals’ efforts to conserve them, and the resulting cycles of resource loss and gain [10, 42, 43]. Job demands—such as workload, time pressure, and emotional strain—function as stressors that require employees to expend their limited physical, emotional, and cognitive resources intensively [44, 45].

According to COR theory, high job demands place significant strain on employees’ psychological resources and may lead individuals to continuously evaluate and re-evaluate their work experiences. Under conditions of stress—when resources are threatened or depleted—individuals are motivated to protect their remaining resources or acquire new ones [14]. However, when the resource depletion and stress generated by intense job demands spill over into non-work time, individuals experience difficulty in achieving psychological detachment from work, which in turn triggers work-related rumination [46, 47]. In the context of healthcare, factors such as critical decision-making responsibilities, high levels of patient accountability, and demanding work pace further increase the likelihood that such rumination becomes chronic. Indeed [12], indicate that when healthcare professionals perceive a threat to their personal resources, they tend to cognitively sustain this threat through rumination, thereby elevating their levels of anxiety.

Although prior research suggests that high job demands increase levels of rumination [48, 49], studies examining this relationship specifically within the healthcare sector remain limited. Existing evidence indicates that job demands among healthcare workers [50] and long working hours among physicians [20] constitute key antecedents that trigger rumination.

A similar mechanism operates with regard to the emotional dimensions of job demands. While emotional demands require sustained empathy and self-regulation, emotional dissonance generates an additional cognitive burden due to the conflict between felt and displayed emotions [29, 30]. This internal conflict and emotional load hinder individuals’ ability to achieve psychological detachment from work, thereby fueling the cycle of rumination. From COR perspective, employees are expected to restore the resources expended during work through recovery processes in their non-work time (e.g., psychological detachment and sleep) [18]. However, rumination is known to prolong psychophysiological activation, thereby impairing mental recovery and sleep quality [51]. In sum, within the COR framework, job demands function as primary factors that threaten and deplete individual resources, whereas rumination constitutes a cognitive response to this threat and represents a critical mechanism that propels individuals into a destructive “loss spiral” [51].

Consequently, the cognitive fatigue induced by workload, together with the internal processes triggered by emotional demands, disrupt employees’ recovery mechanisms and generate adverse long-term effects on psychological health. Based on these considerations, the following hypotheses are proposed:

#### H_4_:

Workload has a positive effect on healthcare professionals’ levels of rumination.

#### H_5_:

Emotional demands have a positive effect on healthcare professionals’ levels of rumination.

#### H_6_:

Emotional dissonance has a positive effect on healthcare professionals’ levels of rumination.

### Rumination and burnout

Rumination may impair the process of psychological recovery by preventing individuals from mentally detaching from work. Work-related thoughts that persist beyond working hours continue to consume psychological resources such as energy and attention [52]. By continuously occupying working memory, rumination erodes fundamental psychological resources such as self-esteem and self-efficacy. As self-esteem declines, individuals become increasingly preoccupied with their failures, thereby creating a vicious cycle—a loss spiral—in which resources are depleted at an accelerating rate [51]. According to COR theory, this process intensifies the cycle of resource loss, ultimately leading to stress and burnout [10].

From COR perspective, resources are expected to be replenished during non-work time; however, individuals who ruminate undermine this recovery process by continuing to expend their cognitive resources on past stressors [52]. Within this framework, rumination—defined as the persistent and involuntary contemplation of negative work-related events and emotions outside working hours—is conceptualized as a “parasitic drain on energy” [18]. Even when the stressor is no longer physically present, individuals continue to engage with it mentally, thereby depleting their existing cognitive and emotional resources [53]. Consequently, individuals begin the next workday with diminished resources, which, as posited by COR theory, may evolve into a chronic burnout spiral over time [42].

Empirical studies consistently demonstrate that work-related rumination is positively associated with burnout, stress, and psychological fatigue [18, 23, 48, 49]. Individuals who repeatedly cognitively process their work experiences tend to exhibit greater emotional exhaustion and higher levels of burnout [54]. Similarly, among healthcare professionals, elevated levels of rumination have been shown to increase burnout and hinder psychological recovery [55]. Particularly in high-responsibility contexts such as healthcare delivery, continued cognitive activation after work is emphasized as a factor that erodes employees’ mental well-being [56]. Moreover, insufficient rest combined with persistent rumination in healthcare settings has been linked to cognitive impairments among nurses, increased workplace accidents, reduced productivity, and, most critically, a rise in medication errors [57].

Conversely, healthcare professionals who are able to achieve psychological detachment from work exhibit lower levels of rumination, enabling more effective restoration of depleted cognitive and emotional resources [58]. Recent evidence further confirms that, in the absence of adequate resource support, rumination renders individuals more vulnerable to burnout [36].

In this regard, rumination is conceptualized not merely as a mode of thinking, but as a mechanism that depletes healthcare professionals’ limited psychological resources and directly triggers burnout. Accordingly, the following hypothesis is proposed:

#### H_7_

Rumination has a positive effect on healthcare professionals’ levels of burnout.

### Mediation role of rumination

The mediating role of rumination in explaining burnout has been suggested in prior research However, some of these studies lack a clearly articulated theoretical framework [20–22], while others draw on diverse theoretical perspectives, including resource allocation theory, the effort–recovery model, the job demands–resources model, and COR theory. For instance [59], in line with resource allocation theory, demonstrated that rumination mediates the relationship between negative emotions and job burnout. Sousa and Neves [60], within the framework of the effort–recovery model, found that emotional rumination mediates the effect of workload on emotional exhaustion and disengagement. In contrast, a study grounded in the job demands–resources model hypothesized that rumination would mediate the cross-lagged effects of work pace and role conflict on burnout; however, this hypothesis was not supported [61]. Drawing explicitly on COR theory [62], identified rumination as a mediator in the relationship between psychological distress and burnout among nurses.

Previous research further indicates that different forms of excessive workload exert spillover effects beyond the workplace, often manifesting as work-related rumination [56]. Moreover, from the perspective of emotional demands and emotional dissonance, rumination—conceptualized as a dysfunctional emotion-focused coping strategy [63]—has been robustly associated with negative affect in the literature [64]. Considering the nature of ruminative thinking, it is critical that employees are not simultaneously exposed to multiple job demands—whether quantitative (e.g., time pressure) or qualitative (e.g., cognitive and emotional demands). As demands intensify, employees are more likely to experience difficulties in goal attainment, which in turn increases their tendency to engage in repetitive thinking processes, ultimately contributing to burnout [65]. Geisler et al. [61] also found that emotion suppression as an emotion regulation strategy contributes to burnout, with rumination exerting an indirect effect in this relationship.

Due to its emotional and cognitive nature, rumination sustains physiological activation and prevents individuals from fully benefiting from the restorative function of non-work time. This process leads to the depletion and insufficient replenishment of resources, a line of reasoning that is also consistent with COR theory [66].

#### H_8_:

Rumination mediates the relationship between workload and burnout.

#### H_9_:

Rumination mediates the relationship between emotional demands and burnout.

#### H_10_:

Rumination mediates the relationship between emotional dissonance and burnout.

**Fig. 1:**
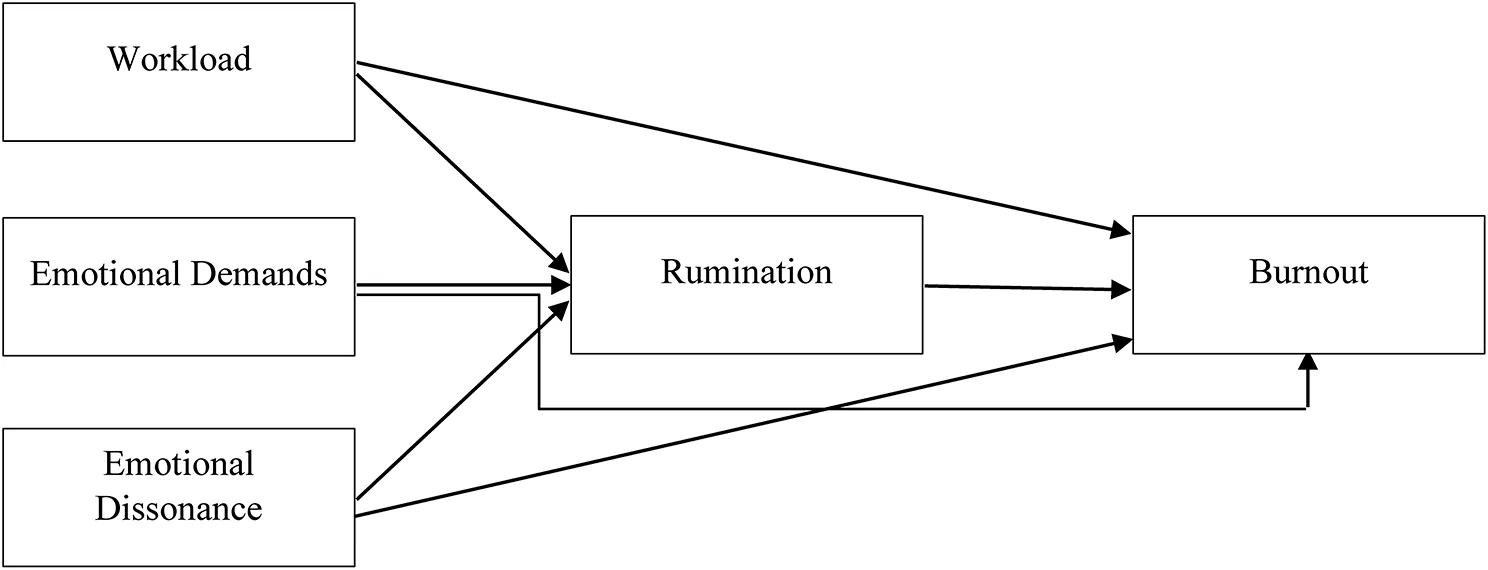
Research model

In line with these hypotheses, the research model is constructed as follows (Fig. 1):

## Method

### Sample and procedure

The population of the study consists of healthcare professionals working in Türkiye. The sample, on the other hand, comprises healthcare professionals employed in the provinces of Istanbul and Eskişehir. Istanbul, as the largest metropolitan city in Türkiye, hosts a wide range of public and private healthcare institutions and represents a setting where healthcare services are delivered intensively. Eskişehir, although a mid-sized city, is home to major public and university hospitals and constitutes an important centre for the active provision of healthcare services. Collecting data from these two distinct urban contexts has contributed to obtaining a more diverse sample capable of representing different working environments within the healthcare sector.

The research data were collected using a convenience sampling method from healthcare professionals working in Istanbul and Eskişehir who voluntarily agreed to participate in the study. The inclusion of participants from different professional groups and units within the healthcare sector enhanced the diversity of the sample, thereby enabling a more comprehensive interpretation of the findings within the healthcare context. The data collection process was carried out exclusively through online surveys. After excluding incomplete or invalid responses, a total of 408 valid questionnaires were retained for analysis. Considering that, in quantitative research within the social sciences, sample sizes of 400 or more are generally deemed sufficient for multivariate statistical analyses [67], the dataset was considered adequate.

An examination of the demographic characteristics of the participants indicates that the majority were female (78.7%), married (59.1%), and employed in the public sector (83.6%). In terms of educational attainment, bachelor’s degree holders constituted the largest group (45.1%), whereas high school graduates represented the smallest group (8.8%). Regarding work experience, 33.1% of participants had between 1 and 10 years of experience, while only 1.0% had 41 years or more. Finally, the occupational distribution revealed that 46.3% of the participants were nurses and 30.4% were physicians (Table 1).

**Table 1:** Sociodemographic characteristics of participants (*N* = 408)

Characteristic	Category	*n*	%
Gender	Male	87	21.3
	Female	321	78.7
Age (years)	≤ 20	4	1.0
	21–30	140	34.3
	31–40	115	28.2
	41–50	129	31.6
	51–60	17	4.2
	≥ 61	3	0.7
Marital status	Single	143	35.0
	Divorced/widowed	24	5.9
	Married	241	59.1
Healthcare-sector work experience	< 1 year	41	10.0
	1–10 years	135	33.1
	11–20 years	122	29.9
	21–30 years	91	22.3
	31–40 years	15	3.7
	≥ 41 years	4	1.0
Educational attainment	High school	36	8.8
	Associate degree	63	15.4
	Bachelor’s degree	184	45.1
	Postgraduate degree	125	30.6
Employment sector	Public	341	83.6
	Private	67	16.4
Profession	Other staff	95	23.3
	Physician	124	30.4
	Nurse	189	46.3

### Measures

Previously validated instruments were used to measure job demands, rumination, and burnout using a 7-point Likert scale (1 = Never to 7 = Always). Evaluations for all scales were based on the arithmetic mean of participants’ item scores.

Job Demands: Participants’ levels of job demands were assessed using the three subdimensions (Workload, Emotional Demands, and Emotional Dissonance) of the Job Demands–Resources Scale developed by Xanthopoulou et al. [68] and adapted into Turkish by Metin [69]. In the adaptation study, the Cronbach’s alpha coefficients were reported as 0.86 for the workload subscale, 0.77 for the emotional demands subscale, and 0.83 for the emotional dissonance subscale. A sample item for Workload is “Do you have too much work to do?”. A sample item for Emotional Demands is “Do you face emotionally charged situations in your work?”. A sample item for Emotional Dissonance is “During your work, how often do you have to express positive feelings toward your clients while you actually feel indifferent?”.

Rumination: Rumination levels were measured using the 10-item Short Form of the Ruminative Response Scale developed by Treynor et al. [70] and adapted into Turkish by Erdur-Baker and Bugay [71]. The internal consistency coefficient (Cronbach’s alpha) of the scale was reported as 0.85 in the adaptation study. A sample item for Rumination is “Think ‘What am I doing to deserve this?”.

Burnout: Participants’ burnout levels were assessed using the 10-item Burnout Scale developed by Malach-Pines [72] and adapted into Turkish by Tümkaya et al. [73]. In the adaptation study, the Cronbach’s alpha coefficient of the scale was found to be 0.91. A sample item for Burnout is “When you think about your work overall, how often do you feel the following? Tired.”.

## Results

### Reliability and validity findings

In the first stage of the analysis, the validity and reliability of the study variables were assessed. For this purpose, the items corresponding to workload within the job demands construct were coded as “W,” emotional demands as “EDe,” and emotional dissonance as “EDis.” The items measuring rumination were coded as “R,” while those measuring burnout were coded as “B.” Within the research model, workload, emotional demands, and emotional dissonance were treated as independent variables; rumination as the mediating variable; and burnout as the dependent variable. The model was then constructed and analyzed using the SmartPLS software.

For the assessment of validity, a factor loading threshold of 0.40 was adopted. In addition, the Average Variance Extracted (AVE) values for each construct were required to exceed 0.50 [74]. Items that did not meet the factor loading threshold were removed from the model, including two items from the workload construct (W1, W3), three items from the emotional demands construct (EDe5, EDe6, EDe7), and one item from the rumination construct (R5). The results obtained following this refinement process are presented in Table 2.

**Table 2:** Findings related to reliability and validity

Variables	Factor Loadings	Cronbach’s Alpha	CR	AVE
Workload		0.701	0.701	0.540
W2	0.736			
W4	0.733			
Emotional Demands		0.776	0.776	0.537
EDe8	0.734			
EDe9	0.755			
EDe10	0.707			
Emotional Dissonance		0.840	0.838	0.512
EDis11	0.745			
EDis12	0.594			
EDis13	0.785			
EDis14	0.781			
EDis15	0.651			
Rumination		0.908	0.907	0.529
R1	0.798			
R2	0.888			
R3	0.776			
R4	0.835			
R6	0.690			
R7	0.630			
R8	0.586			
R9	0.773			
R10	0.469			
Burnout		0.941	0.940	0.616
B1	0.842			
B2	0.889			
B3	0.817			
B4	0.836			
B5	0.778			
B6	0.826			
B7	0.685			
B8	0.626			
B9	0.597			
B10	0.844			

The validity findings presented in Table 2 indicate that all item factor loadings exceed the threshold of 0.40. The AVE values were calculated as 0.540 for Workload, 0.537 for Emotional Demands, 0.512 for Emotional Dissonance, 0.529 for Rumination, and 0.616 for Burnout. As all values are above the recommended threshold of 0.50, the criterion is satisfied. Based on these results, it is concluded that convergent validity has been established.

The remaining indicators in Table 2 pertain to the reliability of the scales. For reliability measures such as Cronbach’s Alpha and Composite Reliability (CR), a threshold of 0.70 is recommended in the literature [74]. In this regard, the reported values—Workload (Cronbach’s Alpha = 0.701; CR = 0.701), Emotional Demands (Cronbach’s Alpha = 0.776; CR = 0.776), Emotional Dissonance (Cronbach’s Alpha = 0.840; CR = 0.838), Rumination (Cronbach’s Alpha = 0.908; CR = 0.907), and Burnout (Cronbach’s Alpha = 0.941; CR = 0.940)—all meet the recommended thresholds, indicating satisfactory internal consistency reliability.

In addition to these findings, discriminant validity was also assessed. For this purpose, the Fornell–Larcker criterion and HTMT (Heterotrait–Monotrait ratio) values were examined. The literature suggests that, for the Fornell–Larcker criterion, the square root of AVE for each construct should be greater than its correlations with other constructs [75], while HTMT values should be below 0.90 [76]. Based on these criteria, the results presented in Table 3 were obtained.

**Table 3:** Discriminant validity findings

	Fornell-Larcker Criterion					HTMT			
Scales	1	2	3	4	5	1	2	3	4
Burnout	**(0.785)**					-			
Emotional Demands	0.517	**(0.733)**				0.511			
Emotional Dissonance	0.452	0.671	**(0.715)**			0.445	0.663		
Rumination	0.684	0.512	0.539	**(0.727)**		0.685	0.507	0.529	
Workload	0.494	0.646	0.508	0.398	**(0.735)**	0.488	0.644	0.508	0.390

The values presented in parentheses (…) represent the square root of the AVE values.

The Fornell–Larcker results presented in Table 3 indicate that the square root of AVE for each construct is greater than its correlations with other variables. In addition, all HTMT values were found to be below the threshold of 0.90, thereby confirming that discriminant validity has been established.

### Hypothesis testing

Before proceeding with hypothesis testing, Common Method Bias (CMB) was assessed using Harman’s single-factor test. In this test, all items related to the study variables are subjected to an exploratory factor analysis within a single pool. If a single-factor structure emerges or if one factor accounts for more than 50% of the total variance, the presence of CMB is inferred [77]. Accordingly, all items measuring Workload, Emotional Demands, Emotional Dissonance, Rumination, and Burnout were analysed together using exploratory factor analysis. The results revealed a six-factor structure with eigenvalues greater than 1. In addition, the total variance explained was 64.656%, while the highest variance explained by a single factor was 34.853%. Since no single factor accounted for more than 50% of the total variance, it was concluded that CMB is not a concern in this study.

The hypotheses were tested using SmartPLS (Partial Least Squares Structural Equation Modeling). The reason for using this approach is that PLS-SEM is less dependent on multivariate normality assumptions, is suitable for models with relatively complex structures and moderate sample sizes, and enables robust estimation of standard errors and significance testing through resampling procedures [74]. In this context, analyses were conducted using a bootstrapping procedure with 5,000 resamples. The results yielded the indicators presented in Table 4. First, the Variance Inflation Factor (VIF) values were examined to assess multicollinearity. The obtained values ranged between 1.502 and 2.755, all below the threshold of 5, indicating that multicollinearity is not a concern [78].

Subsequently, the effect sizes (f²) of job demand variables on Rumination and Burnout were evaluated. Based on Cohen’s [79] criteria, values of 0.02, 0.15, and 0.35 indicate small, medium, and large effect sizes, respectively. The findings indicate that the effect of Rumination on Burnout is large (f² = 0.443), whereas the effect of Workload on Burnout is small (f² = 0.057). Similarly, Emotional Demands (f² = 0.035) and Emotional Dissonance (f² = 0.097) exhibit small effects on Rumination. In contrast, the effects of Emotional Demands and Emotional Dissonance on Burnout (f² = 0.012; f² = 0.001), as well as the effect of Workload on Rumination (f² = 0.004), were found to be below the 0.02 threshold, indicating no meaningful effect [80].

The explanatory power (R²) of the model was also examined, revealing that Burnout and Rumination were explained at levels of 53% and 33%, respectively. Finally, the predictive relevance coefficients (Q²) were assessed. The Q² values were found to be 0.295 for Burnout and 0.155 for Rumination, both exceeding zero, thereby satisfying the recommended criteria for predictive relevance [78].

**Table 4:** Structural model coefficients

Scales			VIF	f^2^	Scales	*R* ^2^	Q^2^
Emotional Demands	➜	Burnout	2.439	0.012	Burnout	0.531	0.295
Emotional Dissonance	➜	Burnout	2.029	0.001	Rumination	0.334	0.155
Rumination	➜	Burnout	1.502	0.443			
Workload	➜	Burnout	2.755	0.057			
Emotional Demands	➜	Rumination	2.356	0.035			
Emotional Dissonance	➜	Rumination	1.850	0.097			
Workload	➜	Rumination	1.747	0.004			

**Fig. 2:**
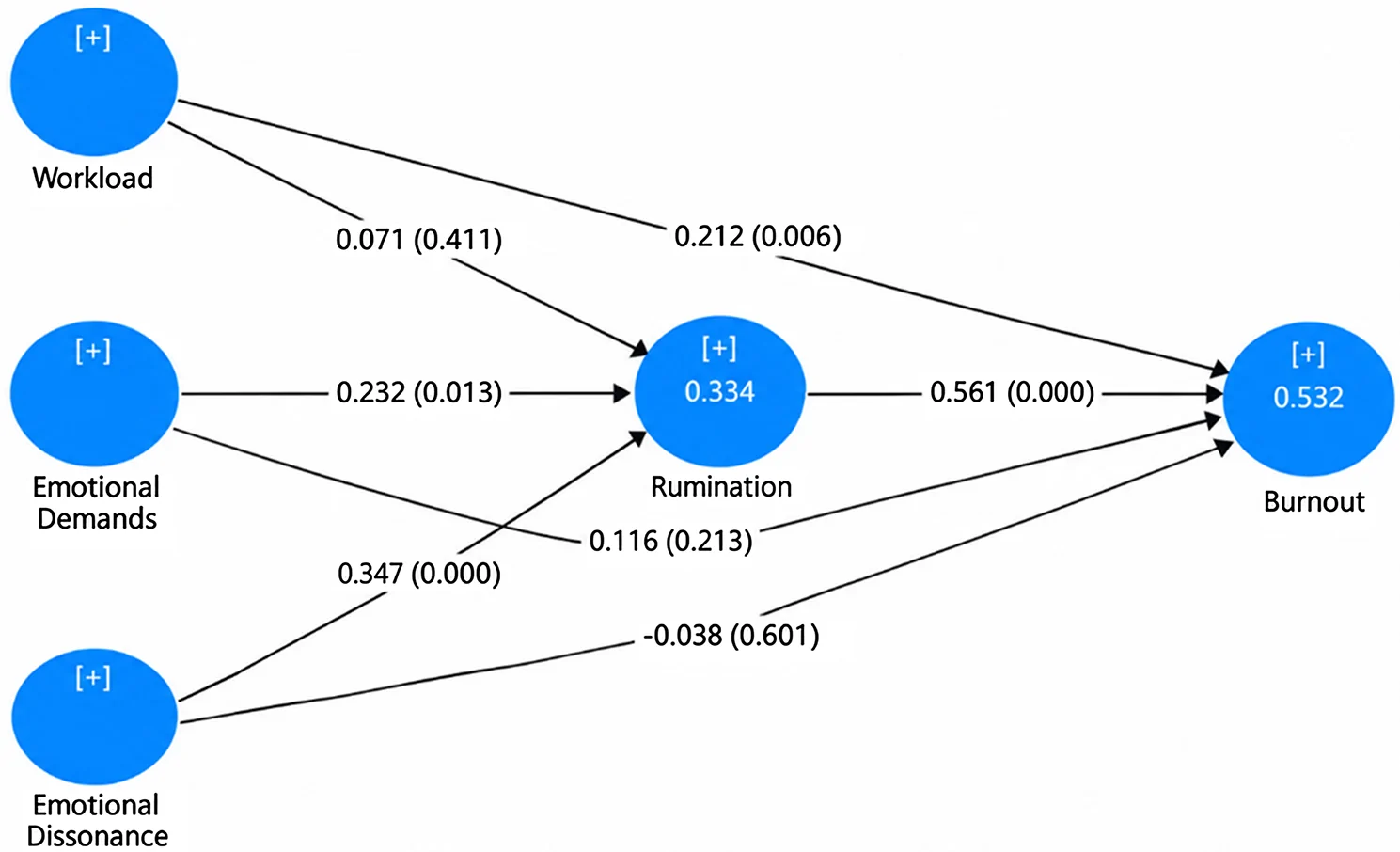
PLS-SEM model

The output of the model estimated using the bootstrapping procedure is presented in Fig. 2. The results of the hypothesis tests obtained within this model—covering both direct and indirect effects—are reported in Table 5.

**Table 5:** Findings related to the hypotheses

Direct Effects	Path coefficient		S.D.	T Statistics	*P* Values	CI
Emotional Demands ➜ Burnout		0.116	0.093	1.244	0.213	-0.066-0.300
Emotional Demands ➜ Rumination		0.232	0.094	2.472	**0.013**	**0.046–0.420**
Emotional Dissonance ➜ Burnout		-0.038	0.073	0.523	0.601	-0.188-0.099
Emotional Dissonance ➜ Rumination		0.347	0.074	4.700	**0.000**	**0.198–0.493**
Rumination ➜ Burnout		0.561	0.063	8.949	**0.000**	**0.450–0.695**
Workload ➜ Burnout		0.212	0.078	2.726	**0.006**	**0.058–0.362**
Workload ➜ Rumination		0.071	0.087	0.822	0.411	-0.111-0.234
Indirect Effects						
Emotional Demands ➜ Rumination➜ Burnout		0.130	0.057	2.281	**0.023**	0.026–0.251
Emotional Dissonance ➜ Rumination➜ Burnout		0.195	0.049	3.949	**0.000**	0.109–0.301
Workload➜ Rumination ➜ Burnout		0.040	0.050	0.798	0.425	-0.060-0.138

Note: The bootstrapping method was used (5000 subsamples)

In the literature, two main approaches are commonly employed to assess mediation effects: the classical criteria proposed by Baron and Kenny [81] and the more contemporary perspective advanced by Zhao et al. [82]. In the present study, the approach of Zhao et al. [82] was adopted. The reason for this preference is that the approach proposed by Zhao et al. (2010) is based on the direct testing of indirect effects using bootstrapping rather than relying on the sequential significance criteria of Baron and Kenny [81] and enables the classification of full, partial, or indirect-only mediation through a decision tree in contemporary mediation analysis. Accordingly, the findings reported in Table 5 were examined, and the direct effects were evaluated first. The results indicate that Emotional Demands (β = 0.116; *p* = 0.213) and Emotional Dissonance (β = -0.038; *p* = 0.601) do not have a statistically significant direct effect on Burnout. In contrast, Workload was found to have a positive and significant direct effect on Burnout (β = 0.212; *p* = 0.006). Furthermore, Emotional Demands (β = 0.232; *p* = 0.013) and Emotional Dissonance (β = 0.347; *p* = 0.000) were found to exert positive and significant direct effects on Rumination, whereas this effect was not observed for Workload (β = 0.071; *p* = 0.411). The final direct effect finding demonstrates that Rumination has a positive and significant effect on Burnout (β = 0.561; *p* = 0.000). Based on these results, hypotheses H1, H5, H6, and H7 were supported, while hypotheses H2, H3, and H4 were rejected.

Regarding the mediation hypotheses, the indirect effects were examined. The findings reveal that Emotional Demands (β = 0.130; *p* = 0.023; CI = 0.026–0.251) and Emotional Dissonance (β = 0.195; *p* = 0.000; CI = 0.109–0.301) have positive and significant indirect effects on Burnout through Rumination. To determine the type of mediation, the Mediation Effect Decision Tree was employed [59], and these effects were identified as full mediation. Accordingly, hypotheses H9 and H10 were supported. In contrast, the indirect effect of Workload on Burnout through Rumination (β = 0.040; *p* = 0.425; CI = -0.060-0.138) was not statistically significant; therefore, hypothesis H8 was rejected.

## Discussion

This study investigates the impact of distinct job demands—workload, emotional demands, and emotional dissonance—on burnout among healthcare professionals, with particular emphasis on the mediating role of rumination within the framework of COR theory. The findings contribute to theoretical understanding and offer actionable insights for promoting the sustainability of healthcare systems and enhancing employee well-being.

This current cross-sectional study found that workload has a direct and positive relationship with burnout (H1). This finding aligns with recent public health evidence emphasizing that healthcare professionals rapidly deplete their energy resources under conditions of chronic time pressure and excessive task demands [8, 38]. From a COR perspective [11], workload generates a “primary resource loss,” which, when not compensated within the workday, may contribute to a burnout spiral.

In contrast, the non-significant direct associations of emotional demands (H2) and emotional dissonance (H3) on burnout constitute an unexpected yet partially consistent finding with prior literature. Job demands such as emotional demands and emotional dissonance are frequently encountered as inherent aspects of the profession, and over time, employees may develop coping strategies through accumulated professional experience. Healthcare professionals may perceive intense emotional interactions as an inevitable part of their role, potentially developing a higher tolerance or employing adaptive strategies like cognitive reappraisal and “deep acting” to mitigate direct strain [30, 83–86]. Importantly, this does not imply that emotional demands are harmless; rather, it suggests that their impact operates not through immediate energetic depletion but through a more complex cognitive mechanism—namely, rumination.One notable finding is that rumination fully mediated the relationship between emotional demands (H9), emotional dissonance (H10), and burnout. This result indicates that emotional stressors do not directly exhaust healthcare professionals in isolation; instead, their impact emerges through the repeated cognitive processing of these stressors during non-work time (i.e., rumination). Although COR theory has long posited that resource loss begets further loss, the specific psychological mechanisms through which this process unfolds have remained largely unclear [13]. By positioning rumination as a “cognitive engine” within the COR framework, this study provides evidence consistent with the proposition that rumination functions as a cognitive mechanism that sustains and accelerates resource loss cycles even after the stressor has ceased [12, 59, 66].

The present findings extend the literature by positioning rumination, within the COR framework, as a paradigmatic “cognitive engine” (momentum mechanism) that sustains and accelerates resource loss cycles. In this context, rumination functions as a momentum mechanism that maintains resource depletion at the cognitive level even after the stressor has ceased to exist, thereby preventing the interruption of the loss spiral. The parasitic nature of rumination—its tendency to consume resources while simultaneously hindering their replenishment [66]— is consistent with the findings of the present study. Specifically, findings indicating that repetitive negative thinking depletes limited cognitive and emotional resources and is associated with burnout (H7), as well as evidence showing that the sustained perception of resource threat through rumination erodes psychological resilience, further substantiate this mechanism [12, 59]. Accordingly, the results suggest that burnout is not solely related to the nature of the job itself, but also to the cognitive spillover of work experiences into non-work time. In particular, the finding that emotional demands is associated with higher levels of rumination (H5) and relates to burnout through this pathway aligns theoretically with prior studies emphasizing emotion suppression, negative affect, and psychological distress [22, 59, 62]. While emotion suppression reflects how individuals regulate their responses to emotional experiences, emotional demands represent the emotional load imposed by the job, and psychological distress and negative affect can be considered outcomes of this burden. Moreover, the significant association between emotional dissonance and rumination (H6) indicates that the internal conflict generated by surface acting continues to be cognitively processed even after working hours. This, in turn, hinders psychological detachment and systematically disrupts recovery processes, pointing to a critical underlying mechanism [64]. Consequently, the persistence-generating nature of rumination appears to extend the impact of job demands into non-work time, thereby deepening the burnout process [65].

In contrast, the non-significant effect of workload on rumination (H4 and H8) suggests that different types of job demands influence burnout through qualitatively distinct psychological pathways. While physical and temporal demands contribute to burnout primarily through direct “energy depletion,” emotional demands and dissonance exert their influence indirectly by generating “cognitive preoccupation” [19, 59, 61]. This distinction is also consistent with the nature of tasks: physical demands are typically bounded and close-ended processes with a clear beginning and end, allowing for mental closure once completed. In contrast, emotional demands inherently require meaning-making, thereby creating a cognitive residue that complicates psychological detachment [18]. This finding suggests that resource loss spirals in COR theory are not only determined by the magnitude of loss but also by the duration for which the loss remains cognitively “open” [87]. Accordingly, while workload may function as a challenge stressor—consuming resources but dissipating relatively quickly—emotional demands operate more as hindrance stressors, sustaining rumination and extending resource depletion into non-work time [24, 88].

Finally, when considered jointly within the Challenge–Hindrance framework and COR theory, these findings point to a significant theoretical implication. Emotional demands may be experienced as a “challenge,” and even serve a motivational function, depending on the individual’s available resources and coping strategies [88, 89]. However, once ruminative thought processes are activated, these demands may be more likely to be experienced as hindrance stressors, thereby sustaining resource loss spirals [11, 53]. In this sense, rumination should be conceptualized not merely as an outcome variable, but as a critical threshold mechanism that may alter how stressors are experienced, thereby contributing to the initiation and maintenance of resource loss spirals over time.

Healthcare professionals constitute a critical human resource for the sustainability of public health systems. From a public health perspective, these professionals are not only central to individual patient care but also play a fundamental role in disease prevention, the continuity of healthcare services, and the maintenance of system capacity [1, 3]. Ensuring that this essential workforce can perform its duties effectively and sustainably requires a clear understanding of the mechanisms that deplete their psychological and cognitive resources [11]. Higher levels of job demands in the workplace may contribute to resource loss among healthcare proffesionals, thereby negatively affecting the functioning of health systems. In particular, ruminative processes—characterized by repetitive, cyclical, and unresolved thinking about daily work-related events—extend this depletion beyond working hours and accelerate the exhaustion process [18, 19]. In this regard, the findings obtained from testing the proposed research model highlight two critical and directly actionable domains for healthcare management and policy-makers.

First, considering the direct effect of workload, optimizing staffing levels, clarifying job roles, and structuring rest breaks in alignment with biological rhythms and recovery needs should be treated as primary priorities. This represents a primary prevention strategy aimed at interrupting the “resource loss spiral” within the workplace itself [11]. Second, the “full mediation” effect of emotional demands through rumination underscores the vital importance of equipping employees with skills that facilitate psychological detachment from work [19]. Interventions such as mindfulness-based stress reduction programs, cognitive-behavioral approaches, and institutional psychological support units can play a critical role in breaking the rumination cycle and preventing burnout. Public health authorities should therefore develop policies that not only ensure the physical safety of healthcare workers but also safeguard their “right to mental recovery” during non-work time [55, 90]. This is particularly important given that insufficient organizational support mechanisms and the absence of positive reinforcements may intensify cognitive burden and render employees more vulnerable to burnout [36].

## Limitations and future research

Despite its theoretical and empirical contributions, this study has several limitations. First, the cross-sectional design limits the ability to establish definitive causal relationships; therefore, the findings should be interpreted as associations rather than causal effects. Future research employing longitudinal designs or daily diary methods would provide deeper insights into the cumulative effects of job demands and rumination over time. Second, the occupational distribution of the participants (46.3% nurses, 30.4% physicians and 23.3% other staff) may not be fully representative of the general healthcare workforce in the wider population. Future studies should employ more balanced or stratified sampling strategies to improve the representativeness of the sample across occupational groups. Future studies using more balanced or stratified sampling designs need to be done to overcome this limitation.

Third, the reliance on self-report data introduces the potential for social desirability bias, as participants may tend to present themselves in a more favorable professional light. This limitation could be mitigated in future studies by incorporating multiple data sources, such as supervisor evaluations or biometric indicators of stress (e.g., cortisol levels). In this context, future studies are recommended to employ temporal separation of measurements and the use of multiple data sources. Moreover, although precautions were taken to address common method bias (CMB) in the present study and Harman’s single-factor test indicated that common method bias was not a concern, future studies may also benefit from employing a measured marker variable or an unmeasured latent method factor approach.

## Conclusion

This study provides a comprehensive examination of the role of job demands—workload, emotional demands, and emotional dissonance—in the burnout process among healthcare professionals, as well as the critical mechanism of rumination within the framework of COR theory. The findings suggest that burnout in the healthcare sector is not merely a form of physical fatigue, but rather a chronic process of cognitive resource depletion that extends into non-work time.

From a public health perspective, healthcare management should not only focus on workforce planning but also prioritize institutional interventions that safeguard employees’ “right to mental recovery,” such as mindfulness-based practices and peer-support mechanisms. Importantly, responsibility for enabling psychological detachment should not rest solely with individual clinicians. Healthcare organizations and local clinical leaders should develop supportive workplace policies, staffing practices, and supervisory norms that allow employees to disengage from work outside working hours without fearing negative consequences for their jobs, professional standing, or patient care. Ultimately, protecting the mental health of healthcare professionals is a shared organizational and policy responsibility and a fundamental prerequisite for building a sustainable and resilient public health system.

## Data Availability

The datasets used in the current study are available from the corresponding author upon reasonable request.
